# Supplementary material for: Diverse patients’ attitudes towards Artificial Intelligence (AI) in diagnosis
Source: PLOS Digit Health. 2023 May 19;2(5):e0000237. doi: 10.1371/journal.pdig.0000237 (PMC10198520; doi:10.1371/journal.pdig.0000237)
Supplement: S1 Text — (DOCX) [file pdig.0000237.s001.docx]

YOUGOV’S MATCHING AND WEIGHTING APPROACH FOR THE SAMPLE

Each racial group was matched to a sampling frame on gender, age, and education. The frame was constructed by stratified sampling from the full 2018 American Community Survey (ACS) 1-year sample with selection within strata by weighted sampling with replacements (using the person weights on the public use file).

The matched cases were weighted to the sampling frame using propensity scores. The matched cases and the frame were combined and a logistic regression was estimated for inclusion in the frame. The propensity score function included age, gender, race/ethnicity, years of education, and region. The propensity scores were grouped into deciles of the estimated propensity score in the frame and post-stratified according to these deciles.

Alternate Versions of Dependent Variable

In the analysis, we used a weighted proportion without exclusions (52.9% chose the doctor versus 47.1% for the AI), but including exclusions yielded largely the same results (52.6% for doctor. 47.4% for AI). Similarly, using unweighted data showed little change for data without exclusions (51% doctor, 49% AI) or with exclusions (50.3% doctor, 49.6% AI).
