# Supplementary material for: Diverse patients’ attitudes towards Artificial Intelligence (AI) in diagnosis
Source: PLOS Digit Health. 2023 May 19;2(5):e0000237. doi: 10.1371/journal.pdig.0000237 (PMC10198520; doi:10.1371/journal.pdig.0000237)
Supplement: S1 Table — (DOCX) [file pdig.0000237.s002.docx]

**S1 Table. Logistic Regression Predicting Choice of AI, Full Sample without Exclusions**

|  | **Model 1:  Experimental Conditions** | | **Model 2:  + Demographics** | | **Model 3:**  **+ Trust** | |
| --- | --- | --- | --- | --- | --- | --- |
| Illness Severity | 0.95 | (0.08) | 1.00 | (0.09) | 0.97 | (0.09) |
| Accuracy | 1.48^***^ | (0.12) | 1.49^***^ | (0.13) | 1.44^***^ | (0.12) |
| Incorporation | 0.93 | (0.07) | 0.93 | (0.08) | 0.89 | (0.08) |
| Established | 1.22^**^ | (0.10) | 1.21^*^ | (0.10) | 1.18^+^ | (0.10) |
| Tailored | 1.02 | (0.08) | 1.04 | (0.09) | 1.07 | (0.09) |
| Listens | 1.20^*^ | (0.09) | 1.23^*^ | (0.10) | 1.28^**^ | (0.11) |
| Race Unbiased | 1.10 | (0.09) | 1.10 | (0.09) | 1.13 | (0.10) |
| Financial Unbiased | 1.16^+^ | (0.09) | 1.17^+^ | (0.10) | 1.15^+^ | (0.10) |
| Order | 1.19^*^ | (0.09) | 1.17^+^ | (0.10) | 1.11 | (0.10) |
| Illness Experience | 1.10 | (0.09) | 1.17^+^ | (0.11) | 1.14 | (0.11) |
| Black |  |  | 0.81 | (0.11) | 0.71^*^ | (0.10) |
| Hispanic |  |  | 0.91 | (0.12) | 0.80 | (0.11) |
| Asian |  |  | 0.96 | (0.13) | 0.86 | (0.12) |
| Native |  |  | 1.22 | (0.18) | 1.31^+^ | (0.20) |
| Income |  |  | 1.02 | (0.01) | 1.02 | (0.01) |
| Age |  |  | 0.99^+^ | (0.00) | 0.99^+^ | (0.00) |
| Conservative |  |  | 0.63^***^ | (0.07) | 0.66^***^ | (0.07) |
| Religion Important |  |  | 0.70^***^ | (0.06) | 0.67^***^ | (0.06) |
| Sex |  |  | 1.00 | (0.09) | 0.99 | (0.09) |
| Education |  |  | 1.06^+^ | (0.03) | 1.09^**^ | (0.04) |
| Married |  |  | 0.92 | (0.08) | 0.92 | (0.09) |
| Employed Full-Time |  |  | 1.04 | (0.10) | 1.06 | (0.10) |
| Health |  |  | 1.05 | (0.11) | 0.96 | (0.10) |
| Have Provider |  |  | 1.12 | (0.13) | 1.07 | (0.13) |
| Trust Provider |  |  |  |  | 0.97 | (0.03) |
| Trust Hospital |  |  |  |  | 0.95^+^ | (0.03) |
| Trust AI Companies |  |  |  |  | 1.28^***^ | (0.03) |
| *N* | 2672 |  | 2376 |  | 2375 |  |

Notes: Odds Ratios; Standard errors in parentheses ^+^ *p* < .10, ^*^ *p* < .05, ^**^ *p* < .01, ^***^ *p* < .001
