# Supplementary material for: Diverse patients’ attitudes towards Artificial Intelligence (AI) in diagnosis
Source: PLOS Digit Health. 2023 May 19;2(5):e0000237. doi: 10.1371/journal.pdig.0000237 (PMC10198520; doi:10.1371/journal.pdig.0000237)
Supplement: S2 Table — (DOCX) [file pdig.0000237.s003.docx]

**S2 Table. Logistic Regression Predicting Choice of AI with All Exclusions (Pre-Registered Analysis)**

|  | **Model 1:  Experimental Conditions** | | **Model 2:  + Demographics** | | **Model 3:**  **+ Trust** | |
| --- | --- | --- | --- | --- | --- | --- |
| Illness Severity | 1.10 | (0.12) | 1.24^+^ | (0.15) | 1.25^+^ | (0.15) |
| Accuracy | 1.62^***^ | (0.17) | 1.67^***^ | (0.19) | 1.58^***^ | (0.18) |
| Incorporation | 0.90 | (0.09) | 0.91 | (0.10) | 0.85 | (0.10) |
| Established | 1.32^**^ | (0.14) | 1.29^*^ | (0.15) | 1.29^*^ | (0.15) |
| Tailored | 1.16 | (0.12) | 1.20 | (0.14) | 1.25^+^ | (0.15) |
| Listens | 1.11 | (0.12) | 1.22^+^ | (0.14) | 1.27^*^ | (0.15) |
| Race Unbiased | 1.05 | (0.11) | 1.04 | (0.12) | 1.05 | (0.12) |
| Financial Unbiased | 0.98 | (0.10) | 0.99 | (0.11) | 0.98 | (0.11) |
| Order | 1.21^+^ | (0.13) | 1.18 | (0.13) | 1.11 | (0.13) |
| Black |  |  | 0.81 | (0.14) | 0.68^*^ | (0.12) |
| Hispanic |  |  | 1.20 | (0.21) | 1.01 | (0.18) |
| Asian |  |  | 0.96 | (0.17) | 0.82 | (0.15) |
| Native |  |  | 1.20 | (0.25) | 1.21 | (0.26) |
| Income |  |  | 1.03 | (0.02) | 1.03 | (0.02) |
| Age |  |  | 1.00 | (0.00) | 0.99 | (0.00) |
| Conservative |  |  | 0.66^**^ | (0.10) | 0.71^*^ | (0.11) |
| Religion Important |  |  | 0.64^***^ | (0.08) | 0.61^***^ | (0.08) |
| Sex |  |  | 1.03 | (0.12) | 1.02 | (0.12) |
| Education |  |  | 1.05 | (0.04) | 1.09^+^ | (0.05) |
| Married |  |  | 0.86 | (0.11) | 0.86 | (0.11) |
| Employed Full-Time |  |  | 1.14 | (0.15) | 1.15 | (0.15) |
| Health |  |  | 0.97 | (0.13) | 0.89 | (0.13) |
| Have Provider |  |  | 1.12 | (0.16) | 1.08 | (0.17) |
| Trust Provider |  |  |  |  | 0.94^+^ | (0.03) |
| Trust Hospital |  |  |  |  | 0.97 | (0.04) |
| Trust AI Companies |  |  |  |  | 1.30^***^ | (.045) |
|  |  |  |  |  |  |  |
| *N* | 1538 |  | 1349 |  | 1349 |  |

*Note:* Odds Ratios; Standard errors in parentheses ^+^ *p* < .10, ^*^ *p* < .05, ^**^ *p* < .01, ^***^ *p* < .001. As in primary models in the paper, this analysis excludes respondents that failed an attention check about whether respondent could correctly recall the disease presented in the vignette, and those reporting that they did not understand the vignette (bottom two levels on 10-point scale). In addition, these models exclude respondents who were familiar with the condition (leukemia or sleep apnea, depending on experimental assignment) because of their work in a healthcare setting, or because they, or a close family or friend, has had it.
