# Supplementary material for: Diverse patients’ attitudes towards Artificial Intelligence (AI) in diagnosis
Source: PLOS Digit Health. 2023 May 19;2(5):e0000237. doi: 10.1371/journal.pdig.0000237 (PMC10198520; doi:10.1371/journal.pdig.0000237)
Supplement: Attachment — Submitted filename: Response to reviewers.12.3.22.docx [file pdig.0000237.s004.docx]

December 3, 2022

Editors, PLOS Digital Health

RE: PDIG-D-22-00059 - EMID:6288f71332e9c55f

Dear Editors,

Thank you for the opportunity to revise our manuscript, titled “Diverse Patients’ Attitudes Towards Artificial Intelligence (AI) in Diagnosis PLOS Digital Health.” We appreciate the time and attention of the three peer reviewers, and have carefully reviewed and addressed their comments as follows. Below those, we also acknowledge our compliance with each journal requirement listed in your correspondence.

~ ~ ~

**Reviewer #1, General:** This interesting paper provides the results of a survey of a diverse sample of individuals regarding their likelihood of responding to the use of artificial intelligence in medical care. Working with a survey firm, the authors identified a sample with up-weighted representation designed to ensure sufficient representation of under-represented groups. Participants were asked to indicate whether they would follow the recommendation of an artificial intelligence-facilitated system, under various conditions of physician encouragement, indication of personalization, description of unbiased results, and other conditions. Results suggested a variety of attitudes differing across ethnic groups, political beliefs, religious beliefs, and others.

This paper uses an interesting approach to address an important problem. By focusing on specific questions regarding types of patient responses, the survey addresses the key question of how patients might respond to specific scenarios. As the mixed results across multiple scenarios and groups suggest a complex variety of responses that might require further work to disentangle, this paper opens the door for substantial future work. As interesting as this paper might be, multiple revisions are needed to make this paper more appropriate for publication.

**Response:** We appreciate the summary and engagement with our work. For clarity, we would explain that our primary outcome variable was whether the mock-patients would select to receive advice from such an AI versus a human specialist. Our primary outcome was not “whether they would follow the recommendation of an artificial intelligence-facilitated system.”

**Reviewer #1, Comment 1.** The paper mentions a qualitative study that was used to inform the subsequent survey, but few details are provided regarding the conduct and analysis of the study. Specific description of how the pre-study influenced the subsequent survey would be most useful.

**Response:** Thank you. We have now taken advantage of the journal’s lack of length limitation to provide greater explanation of our methods and findings from the qualitative study.

**Reviewer #1, Comment 2.** The use of weighting and unweighted analyses needs a bit of clarification. Given that the initial sample was weighted, it seems as if an "unweighted" analysis would involve correction of the collected sample back to represent underlying population distributions. It would help to define this analysis explicitly.

**Response:** We appreciate the opportunity to clarify that the original sample was NOT weighted to represent any particular population. Rather that sample was intentionally designed to create a critical mass of respondents in key racial/ethnic groups, so we could have statistical precision to measure any differences in their responses. We then apply weights to approximate the U.S. population for other analyses. We have now made changes to the abstract to clarify our approach.

**Reviewer #1, Comment 3.** The experimental conditions could use some clarifications. For accuracy, listens, tailored, racial, and financial, I assume that the difference is that the prompt is given or not. This should be clarified.

**Response:** Yes, as shown in Table 1B, when these variables were set to “Level 1” the vignettes had “no description” of those issues – the text in Level 2 was not given. We have further revised Table 1B to clarify, and we have added a sentence to the manuscript to emphasize that the vignettes were silent as such.

**Reviewer #1, Comment 4.** The links between the text and the tabular results are a bit hard to interpret. It seems clear that the results in the first paragraphs under "effects of experimental manipulations" are tied to Table 3, but this isn't as clear as it might be. Clearer mention of specific results, etc. might be needed - for example, the description of "clearly listening" might be interpreted as relating to "tailored" or "listens". This should be clarified. Textual errors ("Error! reference source not found") exacerbate this difficulty.

**Response:** We have added clarifications to the text to refer more explicitly to the tables and fix textual errors.

**Reviewer #1, Comment** **5.** The data sharing statement does not adhere to current norms. Given that this is a survey, there's no reason why the exact scenarios, the survey response data, and the analysis code can't be published and shared publicly.

**Response:** We have now provided all of this information in the supplemental materials or Open Science Framework, osf.io/9y26x.

~ ~ ~

**Reviewer #2:** Thanks for the opportunity to read this article, where the authors present very interesting evidence on the acceptability and trustworthiness of AI in the clinical context. Here's some suggestions on parts of the paper that could be improved by the authors. The introduction of the paper is nice and clear and does a good job to introduce and contextualise the current debate on AI in healthcare, but I thought that the authors could do a bit more to present their specific contribution on this issue. This is done partially at the start of the section on findings and implications, but could be anticipated at the end of the introduction. I think this could highlight more explicitly the key contribution of the author and how they add on current discussions.

**Response:** Thank you, we have added a more explicit statement of our contribution, emphasizing both our systematic manipulation of the vignettes to explore issues of accuracy, race, bias, etc., and our unique sampling design which allows us to estimate racial/ethnic differences.

**Reviewer #2:** The author explain how they've carried out both qualitative and quantitative research and they discuss the former as pre-study. I think it would be helpful if the authors could say more on why they went with both qualitative and quantitative and how the two types of results were integrated and weighted. This is partially discussed in the methodological section on statistical analysis, but I think it would be helpful if the authors could expand on the more general rationale behind their methodological choices.

**Response:** As with our response to Reviewer #1, Comment 1, we have buttressed our discussion of the qualitative study and methodological choices.

**Reviewer #2:** A more specific point on the findings of the authors, where I would suggest that the authors spend a bit more time, is about the specific role that the AI plays in the clinical vignettes. The authors mention towards the end of the paper that the inclusion of physician in treatment decision didn't effect uptake, but I wonder: are all situations/vignettes discussed by the authors such that the AI determines on its own the treatment decision? Is this a situation that can be easily compared to one where a physician relies on the AI and other tools? What do the authors think about differences across the spectrum? I wonder how much of the lack of acceptability of AI rests on deferring judgment and trust to machines completely, I wonder if similar issues of acceptability would show if judgment were fully deferred to PET scanning or other technologies.

**Response:** We have expanded the Findings and Implications section to explain, as follows. We expected greater resistance to AI where the ultimately treatment decision is simply outsourced to this machine. In contrast, we expected that patients would be more accepting of medical AI in the half of the vignettes where AI was presented as merely an input to their trusted human physician’s ultimate decision.

**Reviewer #2:** A final point where I would be interested to hear more from the authors concerns the concepts of acceptance and trustworthiness. What is the difference between the two, if any? Do the authors approach and use the two concepts as synonyms or are they the same? To me, it seems like acceptance is an issue that is less grounded on social interactions, which is rather the context where trust emerges. I mention this as a possible distinction because I wonder if the ethnic and socio-economic background of participants can have an effect on trustworthiness, rather than acceptance. I also wonder whether trustworthiness is more about the relationship established between users, infrastructures, technologies, practitioners, rather than the certainty and accuracy of the technology, which maybe plays a more important role for acceptance. See also recent work in philosophy of science on trust and trustworthiness as potential sources of material on these issues:

<https://www.journals.uchicago.edu/doi/10.1093/bjps/axs007>

<https://link.springer.com/article/10.1007/s11229-018-01913-z>

**Response:** Thank you. We have added a sentence to the background section and cited these two sources. Moreover, we would note that we measure Trust in Provider, Trust in Hospital, and Trust in AI Companies, but only find the latter to be associated with AI uptake. We have also added a sentence to the Findings discussion to emphasize that, we found that generalized trust in AI companies is nearly as important for AI uptake as the disclosure that this particular AI is proven more accurate that human specialists.

~ ~ ~

**Reviewer #3:** In this paper, the authors conducted two experiments, interviews with patients in clinical vignettes and a survey from a diverse population, towards the selection of AI versus human physicians. The authors found that a primary care physician’s explanation about AI’s superior accuracy, a primary care physician’s gentle push towards AI, and a listening patient experience would increase the acceptance of AI in clinics. On the other hand, disease severity and other manipulations would not affect the acceptance of AI. Further, the preference of selecting AI in clinic varies among racial groups, Native Americans > European Americans > Africa Americans. Also, those population groups including senior group (with an older age), politically conservative, and viewing religion, are less likely to choose AI.

The authors conducted the qualitive study with 24 patients (most of them are over 50 years old) from Tucson Arizona, and the quantitative survey using the internet.

**Response:** Thank you for your thoughtful review of our paper.

**Reviewer #3:** Qualitive study: Major: The sample size (a total of 24 patients, 10 Whites, 8 Hispanics, 3 Blacks, 2 Native Americans, and 1 Asian) collected in the study is relatively small to make solid conclusions. || Another weakness is the age distribution of these 24 patients, as mentioned in the paper, “ages ranged from 19 to 92 years, with most over 50”. The attitude of these patients may partially relate to the fact that AI was not significantly powerful/trustworthy back to 1970s. || Make a concession, even the sample size is adequate, as mentioned in the paper “all patients are from Tucson, AZ”, it would be better to discuss and mention that the result and conclusion would be valid and appliable in the town of Tucson, Arizona.

**Response:** We agree that the sample size is small, and have revised the manuscript to emphasize that we do not make conclusions on the basis of this qualitative data alone. Our quantitative data confirm the reviewer’s suggestion that older patients may have greater resistance to AI, a point that is discussed in the paper. Note, however, that older patients are also disproportionately likely to use healthcare, which makes our over-representation of such patients (recruited from our clinics) appropriate for understanding perspectives of actual patients. Finally, since we do not claim any particular conclusions from the qualitative data alone, we do not claim generalizability to all of Tucson, Arizona much less to other populations. However, the national, quantitative study is designed specifically to support generalization to the national U.S. population. The qualitative study is best interpretated as merely the groundwork for that later study. We have added a new paragraph to the “Strengths and Limitations” section to address these points.

**Reviewer #3:** Minor: The definition of senior group (who were older), politically conservative, and viewing religion could be clearer. In the second paragraph of Page 6, it would be better to update some phrases. For examples, update “several” to “XX%” or xx out of 24[.]

**Response:** We have revised the Results section of the manuscript to explain that age is modelled year-by-year as a linear effect, so there is no defined senior group. In the Quantitative Survey Methods Section, with the Characteristics of Respondents subsection, we have revised the manuscript to explain how the political conservative and religion variables were measured and analyzed (providing the text of the Likert scales parenthetically).

As for the paragraph of Page 6, which is part of our qualitative interviews, although we appreciate the reviewer’s perspective, we would respectfully decline this suggestion to quantify our qualitative data. Given our small sample size and the convenience sampling approach, we do not believe numerical proportions are useful to characterize our findings, and instead may mislead reviewers to make inferences about larger populations, which are more precise than warranted. (We would normally present confidence intervals for any such outcome estimates, as we do for the large quantitative study, but the assumptions are not satisfied here.) If the editors feel strongly on this point, please let us know.

**Reviewer #3:** Quantitative study, Major: The race and ethnicity study reported a significant difference between race or ethnicity, black respondents had lower odds of selecting AI (OR=.73, CI:.55-.96, p=.023) and Native Americans had higher odds of selecting AI (OR: 1.37, CI: 1.01-1.87, p=.041) than White respondents. A major factor needs to be considered is the composition of each racial population group. As mentioned in the paper, each unit increase in education improves the chance of selecting AI providers. Therefore, compared with the white group, the lower odds of selecting AI in the black group may be due to the lower education level. Therefore, an in-depth study of the difference would be necessary to answer where it come from. Another factor is genetical difference between racial groups [1,2], which has been shown to have impact in different phenotypes. It would be better to discuss such point in the discussion section. || Similarly, for other factors like sex, Religion Important, Age, etc., their group composition should be further investigated. [1]: Martin, A.R., Kanai, M., Kamatani, Y., Okada, Y., Neale, B.M. and Daly, M.J., 2019. Clinical use of current polygenic risk scores may exacerbate health disparities. Nature genetics, 51(4), pp.584-591. [2]: Gao, Y. and Cui, Y., 2020. Deep transfer learning for reducing health care disparities arising from biomedical data inequality. Nature communications, 11(1), pp.1-8.

**Response:** Thank you, we have created a new table (2B) providing descriptive statistics divided by race. We have also added text to the introduction and discussion, explaining that we do not suggest that these differences are biologic or genetic, but rather more likely reflect historical experiences with the healthcare system and structural racism.

~ ~ ~

**Journal Requirements: 1.** Please amend your detailed Financial Disclosure statement. This is published with the article. It must therefore be completed in full sentences and contain the exact wording you wish to be published.

a. Please clarify all sources of funding (financial or material support) for your study. List the grants (with grant number) or organizations (with url) that supported your study, including funding received from your institution.

b. State the initials, alongside each funding source, of each author to receive each grant.

c. State what role the funders took in the study. If the funders had no role in your study, please state: “The funders had no role in study design, data collection and analysis, decision to publish, or preparation of the manuscript.”

d. If any authors received a salary from any of your funders, please state which authors and which funders.

**Response:** We have complied with all of these requirements.

**Journal Requirements: 2.** Please send a completed 'Competing Interests' statement, including any COIs declared by your co-authors. If you have no competing interests to declare, please state "The authors have declared that no competing interests exist". Otherwise please declare all competing interests beginning with the statement "I have read the journal's policy and the authors of this manuscript have the following competing interests:"

**Response:** Done.

**Journal Requirements: 3.** In the online submission form, you indicated that your data will be submitted to a repository upon acceptance. We strongly recommend all authors deposit their data before acceptance, as the process can be lengthy and hold up publication timelines. Please note that, though access restrictions are acceptable now, your entire data will need to be made freely accessible if your manuscript is accepted for publication. This policy applies to all data except where public deposition would breach compliance with the protocol approved by your research ethics board. If you are unable to adhere to our open data policy, please kindly revise your statement to explain your reasoning and we will seek the editor's input on an exemption. Please be assured that, once you have provided your new statement, the assessment of your exemption will not hold up the peer review process.

**Response:** We have now done so on the Open Science Framework.

**Journal Requirements: 4.** Please provide separate figure files in .tif or .eps format and remove any figures embedded in your manuscript file. Please also ensure that all files are under our size limit of 10MB. For more information about how to convert your figure files please see our guidelines: https://journals.plos.org/digitalhealth/s/figures

**Response:** We have done so. We have left the titles and notes for each figure in the manuscript.

**Journal Requirements: 5.** We notice that your supplementary materials are included in the manuscript file. Please remove them and upload them with the file type 'Supporting Information'. Please ensure that each Supporting Information file has a legend listed in the manuscript after the references list.

**Response:** We have done so.

**Journal Requirements: 6.** Please review your reference list to ensure that it is complete and correct. If you have cited papers that have been retracted, please include the rationale for doing so in the manuscript text, or remove these references and replace them with relevant current references. Any changes to the reference list should be mentioned in the rebuttal letter that accompanies your revised manuscript. If you need to cite a retracted article, indicate the article's retracted status in the References list and also include a citation and full reference for the retraction notice.

**Response:** We have done so adding several new references in particular.

~ ~ ~

Thank you again for the opportunity to revise this paper. I hope these revisions are acceptable, but do not hesitate to let us know if we can further improve the manuscript.
