# Supplementary material for: Diverse patients’ attitudes towards Artificial Intelligence (AI) in diagnosis
Source: PLOS Digit Health. 2023 May 19;2(5):e0000237. doi: 10.1371/journal.pdig.0000237 (PMC10198520; doi:10.1371/journal.pdig.0000237)
Supplement: Attachment — Submitted filename: RESPONSE TO REVIEWERS.v2.docx [file pdig.0000237.s005.docx]

RESPONSE TO REVIEWERS

~ ~ ~

Reviewer #1: Thanks for the revisions to the paper. This revision is much improved. However, there are a few lingering issue that should be addressed.

The additional clarity regarding the qualitative analysis is particularly useful. However, there is almost nothing said about how the qualitative study influenced the subsequent survey. We are simply told that "These materials were based on the vignettes that we tested in the qualitative phase, with refinements and clarifications based on feedback from those participants." More detail regarding these changes is needed.

Thank you for this suggestion to better link our qualitative and quantitative phases. We have added the following sentences to the paper: “In particular, we designed our experimental manipulations to address specific concerns that we heard in the interviews, when respondents explained why they would prefer a human physician, even if proven to be less accurate than an AI system. For example, to address respondents’ confidence in their own physician’s advice as to whether to use AI, we designed an experimental condition specifically testing a scenario where a primary care physician strongly recommends the AI consult and even offers to make an appointment with the service. For other expressed concerns, such as the role of faith in God, we did not find feasible implementations in the design of our quantitative stimulus.”

This shortcoming is directly related to a point raised by one of the other reviewers: the lack of detail in the discussion of the qualitative results. The authors are not inappropriate in mentioning that the presentation of statistics might lead to some bias. However, the use of terms such as "most" or "several" is arguably worse. In this case, I believe the risk associated with quantifying the results is acceptable, particularly as the bulk of the paper is focused on the results of the qualitative study.

First, Reviewer 1 may be referring to a prior round of reviews, because at present we would emphasize the other two reviewers are now satisfied with the manuscript as it now stands. Second, Reviewer 1 incorrectly suggests that "the bulk of the paper is focused on the results of the qualitative study," while in fact the bulk of the paper is focused on the **quantitative** study, and we have only added to the discussion of the qualitative pre-study at the request of these reviewers. These two misunderstandings may shape Reviewer 1’s request for further revisions.

As to whether we should quantify our qualitative data, this is a methodological choice in which there is some debate among methodologists, and frankly it depends on the researchers' goals. As one researcher argues, “the whole enterprise of seeking to quantify the expression of views within qualitative research is fraught with difficulties.”^[1]^ Another explains that “particiapants’ comments should not be tallied, counted, or otherwise taken out of the context in which the comments originated.”^[2]^

We never intended this pre-study to itself produce generalizable knowledge; it is merely due diligence on the way to the quantitative study. We think that the qualitative results are best understood qualitatively, especially since they are followed by a large robust quantitative study, which is analyzed quantitatively. In practical terms, to convert qualitative statements like “several respondents expressed…” to precise proportions would now require us to recode the qualitative data and resolve disagreements between coders interpretating ambiguous texts. We see no real benefit of doing so, especially not at this late stage, and frankly we lack grant funding to support such re-analysis. We would respectfully decline the suggestion of Reviewer 1.

A few additional points came to mind in the re-reading of this paper:

1. Table 1a: The text presented to respondents is arguably quite biased. The description of the AI case (Med-X) specifically mentions blood and genetic analysis, whereas neither is mentioned in the discussion of the specialist consult. Respondents might legitimately read this as saying that the physician consult might not involve any blood or genetic testing, and thus conclude that the physician's decision might not be well-informed.

Thank you for noting the ways in which we designed our study. To engage with this comment, we have added this sentence to the methods section: “The base case descriptions and the manipulations were designed to make AI appear attractive relative to human decision-makers.” In the discussion section, we added this additional text: “Given the prior literature showing widespread hesitance to use AI, even when proven more reliable than humans, we specifically designed our stimulus and manipulations to counteract that hesitance, emphasizing potential advantages of the technological approach. For example, the description of the AI option (Med-X) specifically mentions blood and genetic analysis, whereas neither is mentioned in the discussion of the specialist human consult. Respondents might infer that the physician consult might not involve any blood or genetic testing, and thus might not be as well informed. In this sense, we are designing a robust test of the ‘robophobia’ hypothesis.”

2. There is a troubling statement in the introduction: "Although initial forays into algorithmic decisions gave rise to similar concerns (26), AI systems may be rigorously designed, tested, and continuously monitored to minimize racial or financial biases in healthcare (27, 28)." Although technically true, this statement lacks important context - many, if not most, systems, are not built this rigorously. As perceptions of rigor and considerations of bias might be particularly important for responses to the survey, this limitation should be acknowledged.

Thank you for the suggestion. In addition to the new material mentioned just above, The limitations section specifically provides that, “We purposefully included hypothetical features in our descriptions of the AI system, when testing a range of manipulations, such as its lack of racial bias or its proven accuracy beyond that of human specialists. However, these traits may or may not be true about any particular AI system.”

3. Statistical comparisons between groups should be added to figure 2B.

Since the paper does not have a figure 2B, and figure 2 already shows confidence intervals, we presume that Reviewer 1 is referring to Table 2B. In the revised manuscript (“Descriptive Traits of Participants Split by Race / Ethnicity.”) In the revised version of the manuscript, we now provide: “Values marked with a † indicate that the proportions or means for this racial group differed significantly (at the p<.05 level) from the means or proportions of observations in the other racial groups.”

~ ~ ~

1. Sim J. Collecting and analysing qualitative data: issues raised by the focus group. Journal of advanced nursing. 1998 Aug;28(2):345-52.
2. Asbury JE. Overview of focus group research. Qualitative health research. 1995 Nov;5(4):414-20.
